# Measurement of protein backbone ^13^CO and ^15^N relaxation dispersion at high resolution

**DOI:** 10.1007/s10858-017-0127-4

**Published:** 2017-09-01

**Authors:** Maxim Mayzel, Alexandra Ahlner, Patrik Lundström, Vladislav Y. Orekhov

**Affiliations:** 1 The Swedish NMR Centre, University of Gothenburg, Box 465, 40530 Göteborg, Sweden; 2 Division of Chemistry, Department of Physics, Chemistry and Biology, Linköping University, 58183 Linköping, Sweden; 3 Department of Chemistry and Molecular Biology, University of Gothenburg, Box 465, 40530 Göteborg, Sweden

**Keywords:** NMR, NUS, IDP, Conformational exchange, Dynamics, Target acquisition

## Abstract

Peak overlap in crowded regions of two-dimensional spectra prevents characterization of dynamics for many sites of interest in globular and intrinsically disordered proteins. We present new three-dimensional pulse sequences for measurement of Carr-Purcell-Meiboom-Gill relaxation dispersions at backbone nitrogen and carbonyl positions. To alleviate increase in the measurement time associated with the additional spectral dimension, we use non-uniform sampling in combination with two distinct methods of spectrum reconstruction: compressed sensing and co-processing with multi-dimensional decomposition. The new methodology was validated using disordered protein CD79A from B-cell receptor and an SH3 domain from Abp1p in exchange between its free form and bound to a peptide from the protein Ark1p. We show that, while providing much better resolution, the 3D NUS experiments give the similar accuracy and precision of the dynamic parameters to ones obtained using traditional 2D experiments. Furthermore, we show that jackknife resampling of the spectra yields robust estimates of peak intensities errors, eliminating the need for recording duplicate data points.

**Electronic supplementary material:**

The online version of this article (doi:10.1007/s10858-017-0127-4) contains supplementary material, which is available to authorized users.

## Introduction

Millisecond protein dynamics is essential for most protein processes including folding, ligand binding, enzymatic catalysis, and allosteric regulation. Nuclear magnetic resonance (NMR) spectroscopy is especially well suited for characterization of protein dynamics since a unique signal is obtained for each nucleus, enabling studies at atomic resolution at nearly native conditions. The parameters that can be determined for a molecule exchanging between two states are the exchange rate (k_ex_), the population of the excited state (p_B_) and the difference in chemical shifts between the exchanging states (Δϖ). These parameters report on kinetics, thermodynamics and structure of the excited state. A number of distinct NMR techniques have been developed for studies of millisecond dynamics and, if the exchange rate is on the order of hundreds of inverse seconds and the population of the excited state is at least 0.5%, Carr-Purcell-Meiboom-Gill (CPMG) relaxation dispersion (RD) is the method of choice (Orekhov et al. 1994; Loria et al. 1999; Sekhar and Kay 2013).

Severe signal overlap often precludes analysis of important peaks in two-dimensional NMR spectra, such as in the ^1^H–^15^N correlation maps typically used in relaxation experiments. The overlap particularly complicates the dynamic studies of large and disordered protein systems. Increase of spectral dimensionality in combination with non-uniform sampling (NUS) has been widely used during the last decade for dramatic improvement of resolution in the spectra. However, applications of NUS for quantitative analysis such as studies of molecular dynamics is only emerging (Matsuki et al. 2011; Mayzel et al. 2014a, b; Long et al. 2015; Oyen et al. 2015; Linnet and Teilum 2016; Stetz and Wand 2016). The method requires caution to avoid biases in the results due to the inherent non-linearity (Schmieder et al. 1997; Hyberts et al. 2014) of many techniques developed for NUS spectra reconstruction.

In this work, we introduce three-dimensional NUS HNCO-based versions of the ^13^CO and ^15^N RD experiments and validate the method of co-processing for unbiased spectra reconstruction. We also present jackknife resampling, a rigorous statistical procedure for determining confidence regions of the extracted parameters without using repeated measurements. Finally, we demonstrate incremental data accumulation with concurrent spectra processing as a tool for monitoring progress of achieving targets on precision of the peak intensities. The new experiments and analysis are illustrated using two protein systems with well understood dynamics on the millisecond time scale: the SH3 domain from the yeast protein Abp1p partially bound to a peptide from the protein Ark1p and the disordered cytosolic domain of the CD79A chain from the B-cell receptor.

## Methods

### Processing of NUS spectra

The RD technique requires accurate measurements of peak intensities in an array of NMR spectra recorded as a function of frequency (ν) of the refocusing pulses in the CPMG sequence. Traditionally, processing and measuring of peak intensities are performed independently for each spectrum. For processing of individual NUS RD spectra we used one of the modern algorithms Iteratively Reweighted Least Squares with Virtual-Echo (IRLS-VE) (Mayzel et al. 2014; Kazimierczuk and Orekhov 2011).

An alternative approach used in this work exploits the fact that positions and line shapes of peaks are invariant to the CPMG frequency. The most general models for signals in the two- and three-dimensional RD experiments are (Korzhnev et al. 2001; Gutmanas et al. 2004):

1a $$S_{{2D}}^{\nu }=\mathop \sum \limits_i ~\alpha _{i}^{\nu }~V_{i}^{H} \otimes V_{i}^{N}$$

1b $$S_{{3D}}^{\nu }=\mathop \sum \limits_i ~\alpha _{i}^{\nu }~V_{i}^{H} \otimes V_{i}^{N} \otimes V_{i}^{{CO}}$$

where the model of the ν-th spectrum on the left is presented as a sum over components enumerated by index *i*. Each component consists of a peak intensity coefficient *α*_*i*_ and two (three) normalized vectors *V* ^*H*^, *V* ^*N*^, and *V* ^*CO*^, which describe positions and line shapes of a peak for ^1^H, ^15^N, and ^13^CO spectral dimensions, respectively; the symbol ⊗ denotes the tensor product operation, which generates a two (three) dimensional peak object from the vectors. The model in Eq. 1 contains a relatively small number of unknowns because vectors *V* are shared between spectra with different CPMG frequencies. The parameters in the model can be obtained with high fidelity from a few NUS measurements by co-processing of spectra obtained for all ν values simultaneously using multi-dimensional decomposition algorithm (co-MDD) (Mayzel et al. 2014a; Hiller et al. 2009; Orekhov and Jaravine 2011). The number of the model parameters, and consequently the minimal amount of the experimental data needed, can be further reduced by additional assumptions about the functional form for the vectors (Long et al. 2015; Jaravine et al. 2006).

### Error estimations with resampling

The most common practice for estimating errors in relaxation dispersion experiments is based on repeating measurements for some of the CPMG frequencies, from which either the global peak intensity error, if number of repeated measurements is small, or per residue intensity error is estimated. Here, we propose jackknife resampling that eliminates the necessity of the duplicate measurements and provides reliable error estimates for individual residues. Hence, the new method allows sampling of the RD at more CPMG frequencies during the same total experimental time, which in turn is beneficial for subsequent relaxation analysis.

Statistical resampling-based analysis is a natural and preferable alternative to the repeated measurements approach when NUS is utilized for spectra acquisition (Isaksson et al. 2013). In the delete-*d* jackknife procedure presented below, a set of realizations is produced from the recorded data by randomly omitting a small fraction of measurements. According to the theory, *d*—the amount of the omitted data should be equal or exceed the square root of *N*—the total number of NUS data points. In our particular case, since the omission must not significantly reduce sensitivity of the spectra and the chances for accurate peak reconstruction, we omitted $~\sqrt N$ points. Strictly speaking, for the delete-*d* jackknife resampling, all possible subsamples $\left( {\frac{N}{d}} \right)=\frac{{N!}}{{d!\left( {N - d} \right)!}}$ have to be computed. This number quickly becomes very sizable and as an approximation, one can take a small random subset from all possible subsamples. The standard errors of the peak intensities that are calculated over the resampling trials must be up-scaled with the so-called inflation factor $~F=~\sqrt {N/d}$. The inflation factor is needed because intensities in the spectra, obtained by deleting *d* out of *N* observations are highly correlated and the regular standard deviation over resampling trials gives underestimated values (Efron 1993).

In the current study, we consistently used 20 different resampling trials by randomly omitting 15–20% of the acquired data points both for 2D and 3D datasets. As a result of the resampling procedure, for each peak at every CPMG field strength a set of 20 intensities were obtained. The standard deviation of the set, up-scaled with an inflation factor, gives an estimate for the peak intensity error. It should be emphasized that, in contrast to the global error usually obtained from the duplicate measurements, the errors estimated by delete-*d* jackknife resampling are individual for every peak and every CPMG frequency. Another way to utilize the power of resampling techniques is to obtain parameters of the exchange for every resampling trial and then perform statistical analysis of these values to estimate the uncertainties. The possible drawback of the later method is two-fold: first down-sampled spectra have slightly lower signal-to-noise ratio and thus the intensity error is higher, second in order to calculate the relaxation parameters for each resampling trial one still needs estimates of the peak intensity errors. For the relaxation analysis we have not observed any significant difference between these two methods (data not shown), though in some complex tasks, for example backbone assignment, (Isaksson et al. 2013) the latter method is the only possible method to access the uncertainty.

### Error estimations with targeted acquisition

**Fig. 1:**
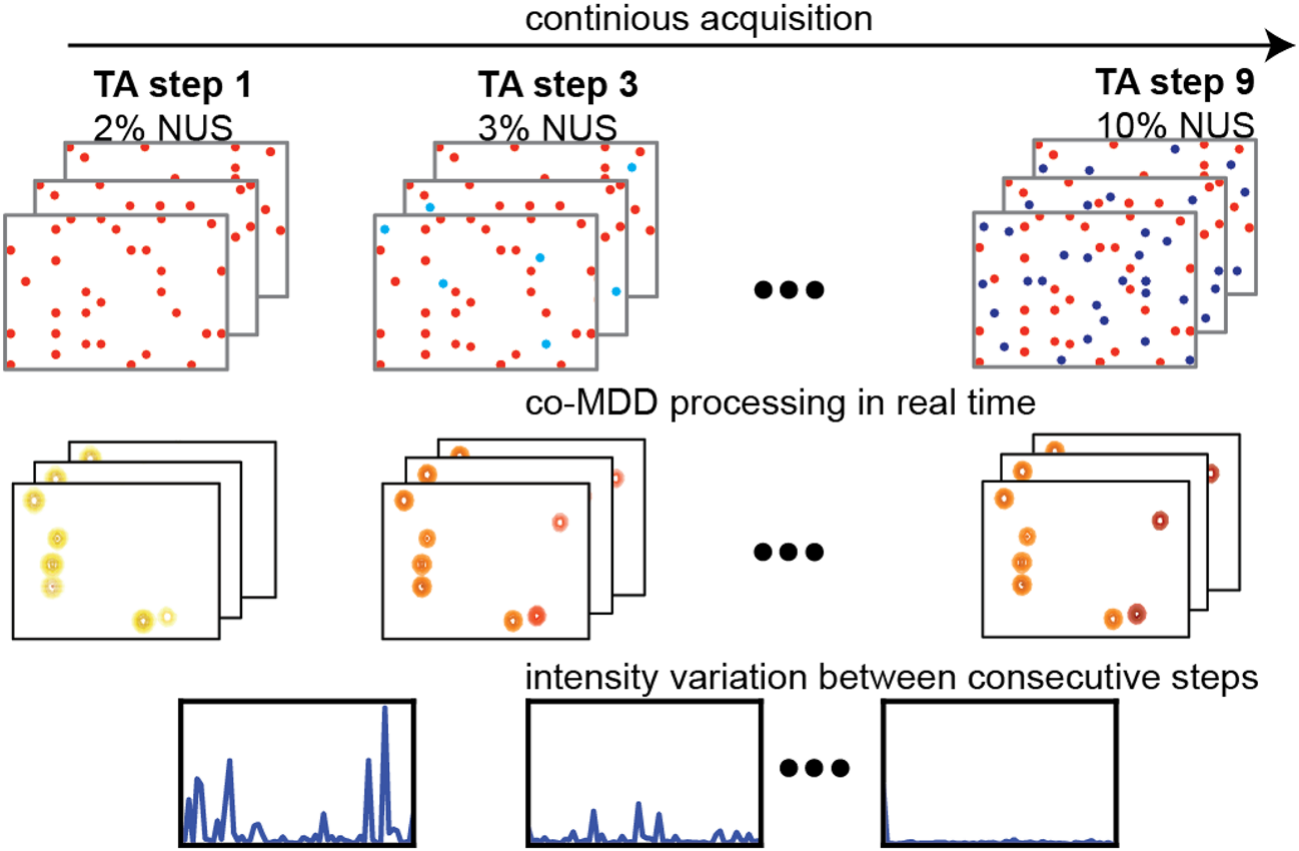
Schematic presentation of the TA procedure for the real-time estimate of the peak intensity precision. The spectra are processed in steps as more and more NUS points are acquired. The precision of individual peak intensities is estimated as the difference between the intensities obtained at two successive steps

An additional advantage of using NUS concerns optimal planning of the RD experiment and addresses the following practical questions: which sparse level and correspondingly how much measurement time is needed for achieving required precision of the measured relaxation rates? Is it feasible to obtain good RD data for a defined set of residues in a particular protein sample? In the traditional approach, the decision about the total measurement time is taken before the experiment starts. Thus, miscalculations are common where either the experiment is too short and RDs of insufficient quality are obtained or the measurement time is too long and spectrometer time is wasted. A solution is found in the concepts of incremental NUS and targeted acquisition (TA) (Jaravine and Orekhov 2006), where the signal processing and statistical analysis are performed in steps concurrently with the experiment (Fig. 1). With such approach, the variation of peak intensities calculated over consecutive steps can be used as a crude estimate of the peak precision at a given time of the experiment.

### Spectra analysis and calculation of dynamic parameters

Recorded spectra were processed with *mddnmr* software using either IRLS algorithm (Kazimierczuk and Orekhov 2011) with Virtual-Echo modification (Mayzel et al. 2014b) or co-processed with co-MDD. For co-MDD the number of iterations and regularization parameter lambda were set to 2000 and 10^−4^, respectively; number of iterations for the IRLS was set to 30. Peak intensities, estimated using the seriesTab script included in the nmrPipe software (Delaglio et al. 1995), were converted into effective transverse relaxation rates ${R_{2,eff}}({\nu _{cpmg}})=\ln \left( {{I_0}/I} \right)/T,$ where $~I$ and *I*_0_ are the intensities with and without the constant time relaxation delay of duration $T$ and ${\nu _{cpmg}}$ is the repetition rate in the CPMG pulse train. Residues with significant chemical exchange (*p* < 0.01) in individual fits were fitted to a global two-state model using the software CATIA (Hansen et al. 2008).

### Protein expression and purification

Uniformly ^13^C/^15^N labeled cytoplasmic domain of human CD79A was produced using an in-house developed cell-free expression system as previously described (Isaksson et al. 2013). Purified and lyophilized CD79A was dissolved to a final concentration of ca. 200 µM in aqueous buffer containing 20 mM NaP_i_ pH 6.8, 1 mM EDTA, Complete EDTA-free protease inhibitor cocktail (Roche), 2 mM DTT, and 10% D_2_O.

Uniformly ^13^C/^15^N labeled SH3 domain from Abp1p was produced and purified as previously described (Vallurupalli et al. 2007). The added peptide was a 17-residue fragment (KKTKPTPPPKPSHLKPK) from the protein Ark1p (Haynes et al. 2007), purchased from EZBiolab. The purified NMR sample was 0.8 mM protein, 50 mM NaP_i_ pH 7.0, 100 mM NaCl, 1 mM EDTA, 1 mM NaN_3_ and 10% D_2_O.

### NMR spectroscopy

All NMR data were acquired at Varian INOVA spectrometers equipped with the room-temperature probe heads at the static magnetic fields of 18.8 T. The sample temperature was 25 °C in all cases. ^15^N- and ^13^CO-CPMG dispersions were acquired by the standard pulse sequences (Lundstrom et al. 2008; Vallurupalli and Kay 2006) as well as using sparse sampling in the three-dimensional HNCO type experiments described above. Experimental details are summarized in Table 1. Sampling schedules, generated using the program *nussampler*, which is part of the *mddnmr* software (Orekhov and Jaravine 2011), had flat random distribution in the relaxation pseudo dimension and exponential matched to 100 ms acquisition in the indirect spectral dimensions. Both classes of experiments were recorded in an interleaved fashion.

**Table 1:** Experimental details

SH3	2D-^15^N-US	2D-^13^CO-US	3D-^15^N-NUS	3D-^13^CO-NUS
Spectral width (Hz)	^15^N 2500	^15^N 2250	^15^N 2250; ^13^C 1400	^15^N 2250; ^13^C 1400
Evolution time (ms)	25.6	28.6	^15^N 13.3; ^13^C 28.6	^15^N 22.2; ^13^C 28.6
N different efficient fields (repetitions)	16 (4)	17 (4)	11 (3)	13 (1)
Interscan delay (s)	3	2.5	3	3
NUS (%)	100	100	8.3	5
Acquisition time (h)	18	16	43	43

CD79A	2D-^15^N -US	2D-^13^CO-US	3D-^15^N-NUS	3D-^13^CO-NUS
Spectral width, Hz	^15^N 2500		^15^N 1800; ^13^C 1400	^15^N 1800; ^13^C 1400
Evolution time, ms	25.6		^15^N 16.7; ^13^C 28.6	^15^N 27.8; ^13^C 28.6
N different efficient fields (repetitions)	16 (4)		11 (4)	12 (2)
Interscan delay, s	3		3	3
NUS, %	100		8.7	8
Acquisition time, h	18		44	63

## Results and discussion

### Pulse sequences for measurements of ^15^N and ^13^CO relaxation dispersions at high resolution

A common problem, even for many small well-folded proteins, is severe spectral overlap that precludes reliable determination of peak volumes, which in turn complicates accurate characterization of protein dynamics for all residues. This problem is of course even more serious for larger or intrinsically disordered proteins. An obvious way of mitigating or reducing this problem is to extend the data to a third dimension. Unfortunately, this increases the measurement time so that a relaxation data set that requires 12 h to record in the normal way would require approximately 1 week recorded in three dimensions, which is prohibitively long. However, if sparse rather than uniform sampling is employed, the data can be recorded in a fraction, perhaps one-tenth, of that time, which would mean that the time requirements would be similar as for the two-dimensional case. With this in mind, we designed three-dimensional pulse sequences for the measurements of ^15^N and ^13^CO CPMG relaxation dispersion. In both these experiments, the flow of magnetization is ^1^H → ^15^N → ^13^CO (t_1_) → ^15^N (t_2_) → ^1^H (t_3_) and they can thus be thought of as HNCO experiments with constant time relaxation delays inserted at appropriate places.

**Fig. 2:**
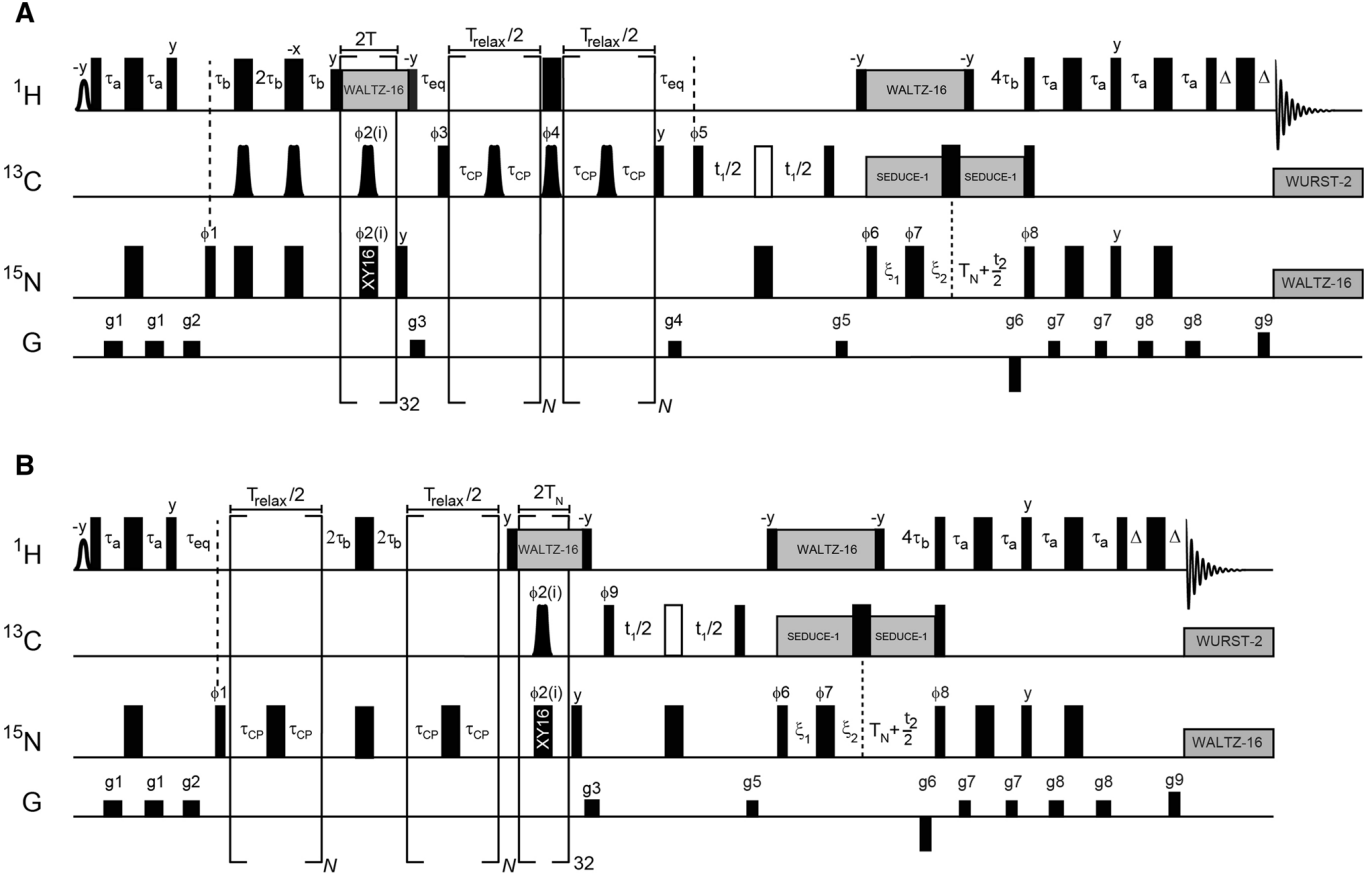
Pulse sequences for measurement of **a** ^13^CO and **b** ^15^N CPMG relaxation dispersions. *Narrow* and *wide rectangles* represent rectangular 90° and 180° pulses, respectively. All pulses are centered at 4.77, 176 and 119 ppm for ^1^H, ^13^C and ^15^N, respectively. The phase of all pulses is x if not specified. The shaped pulse on proton is used to selectively excite the water resonance. A 1.5 ms rectangular pulse was used here. All rectangular 90° pulses on ^13^C are applied at a field strength that yields null at 58 ppm. The 180° pulse represented by an open rectangle is shifted 118 ppm upfield and applied with a field strength that gives a null at 176 ppm. Shaped pulses of duration 450 μs on ^13^C are used to selectively invert or refocus ^13^CO. These are similar to the RE-BURP variety of selective pulses (Geen and Freeman 1991) but have improved inversion profiles (Lundstrom et al. 2008). The simultaneous pulses (applied as a complete train in each scan) during ^15^N→^13^CO transfer have phases ϕ2(i) = 2(x,y,x,y,y,x,y,x,−x,−y,−x,−y,−y,−x,−y,−x) so that both the x and the y components of transverse magnetization are refocused properly in the presence of off-resonance effects and pulse imperfections (Gulltan et al. 1990). The phase cycling is ϕ1 = y,−y; ϕ3 = y,y,−y,−y; ϕ4 = y,−y; ϕ5 = x; ϕ6 = x; ϕ7 = 4(x),4(−x); ϕ8 = x, ϕ9 = x,x,−x,−x, receiver = x,−x,−x,x. Quadrature detection in t_1_ is achieved by incrementing the phase ϕ5 (or ϕ9) by π/2 and in t_2_ by incrementing the phase ϕ6 by π and inverting the gradient g6. For every increment in t_1_ and t_2_ the phases of ϕ5 (or ϕ9) and ϕ8 are incremented by π, respectively. Proton decoupling is achieved by WALTZ-16 at a field of 6 kHz and ^13^Cα decoupling is achieved by SEDUCE-1 that is cosine modulated at 118 ppm (McCoy and Mueller 1992). Decoupling during acquisition employs WALTZ-16 at a field-strength of 1.2 kHz for ^15^N (Shaka et al. 1983) and WURST-2 (bandwidth of 12 ppm, centered at 176 ppm, maximum (rms) B_1_ field of 0.6 (0.4) kHz) for ^13^CO (Kupce and Freeman 1995). The delays are τ_a_ = 2.3 ms, τ_b_ = 1.36 ms, τ_eq_ = 3 ms, T = 10 ms, T_N_ = 14 ms, Δ = 0.5 ms ξ_1_ = max (0, T_N_ - t_1_/2), ξ2 = max (0, t_1_/2 - T_N_). In this scheme, data is recorded in constant-time mode for t_1_ < 2T_N_, whereas magnetization decays for t_1_ > 2T_N_. The gradient-strengths in G/cm (durations in ms) are g1 = 4.0(0.5), g2 = 10.0(1.0), g3 = 7.0(1.0), g4 = −6.0(0.6), g5 = 3.3(0.6), g6 = −30.0(1.25), g7 = 4.0(0.3), g8 = 2.0(0.4), g9 = 29.6(0.125)

Figure 2 shows the pulse sequence used for measurements of ^15^N and ^13^CO dispersions. While the ^13^CO version of the pulse sequence is a straightforward extension of the one already published (Lundstrom et al. 2008), a remark can be made regarding the ^15^N version. At the start of the relaxation delay, the density matrix is equal to 2N_x_H_z_ and it will evolve between anti-phase and in-phase operators in a manner that depends on the number of applied refocusing pulses. Since the different operators have different relaxation rates this introduces artifacts to the dispersion profiles if not addressed. We chose the approach of Palmer and coworkers (Loria et al. 1999) where the time spent as in-phase and anti-phase are equalized, regardless of the number of applied refocusing pulses, by splitting the relaxation delay in half and exchanging in-phase and anti-phase operators in between.

When comparing the sensitivity of the new three-dimensional pulse sequences with the standard two-dimensional ones, there is a difference between pulse sequences designed to measure ^13^CO and ^15^N millisecond dynamics. For ^13^CO, the sensitivity in a single scan is only slightly worse (due to evolution at ^13^CO), implying that the overall sensitivity per measurement time will be about $\sqrt 2$ lower for the three-dimensional version. In two-dimensional experiments that measure ^15^N relaxation dispersions, there is obviously no need to transfer magnetization from ^15^N to ^13^CO and back, implying that the sensitivity losses for the three-dimensional experiment is larger because of relaxation losses during the transfer periods.

The benefits of increased resolution with an extra dimension in the NUS-CPMG are different for different proteins. This was expected and is summarized in Table 2. Proteins, for which the ^15^N-HSQC is highly resolved, such as Abp1p SH3 domain (Drubin et al. 1990), benefit less than proteins with poorly dispersed spectra, such as the intrinsically disordered cytoplasmic domain from CD79A (Isaksson et al. 2013). When peak overlap is not too severe, the 3D pulse sequences can be run in 2D mode, which may allow resolving signals overlapped in either ^15^N or ^13^CO dimensions. However, we did not try this in our work.

**Table 2:** Number of overlapped peaks^a^ for the proteins CD79A and Abp1p SH3 domain in HSQC and HNCO type experiments

Protein	Number of residues	Number of unresolved peaks	
		2D HSQC	3D HNCO
CD79A	63	33	4^b^
Abp1p SH3	59	6	2^c^

^a^As gauged by visual inspection of the spectra drawn at the noise level

^b^Peaks corresponding to the amide groups of residues L13, D17, D32 and L43

^c^Peaks corresponding to the amide groups of residues Y08 and L18

### Accurate relaxation parameters from 2D NUS RD experiments

**Fig. 3:**
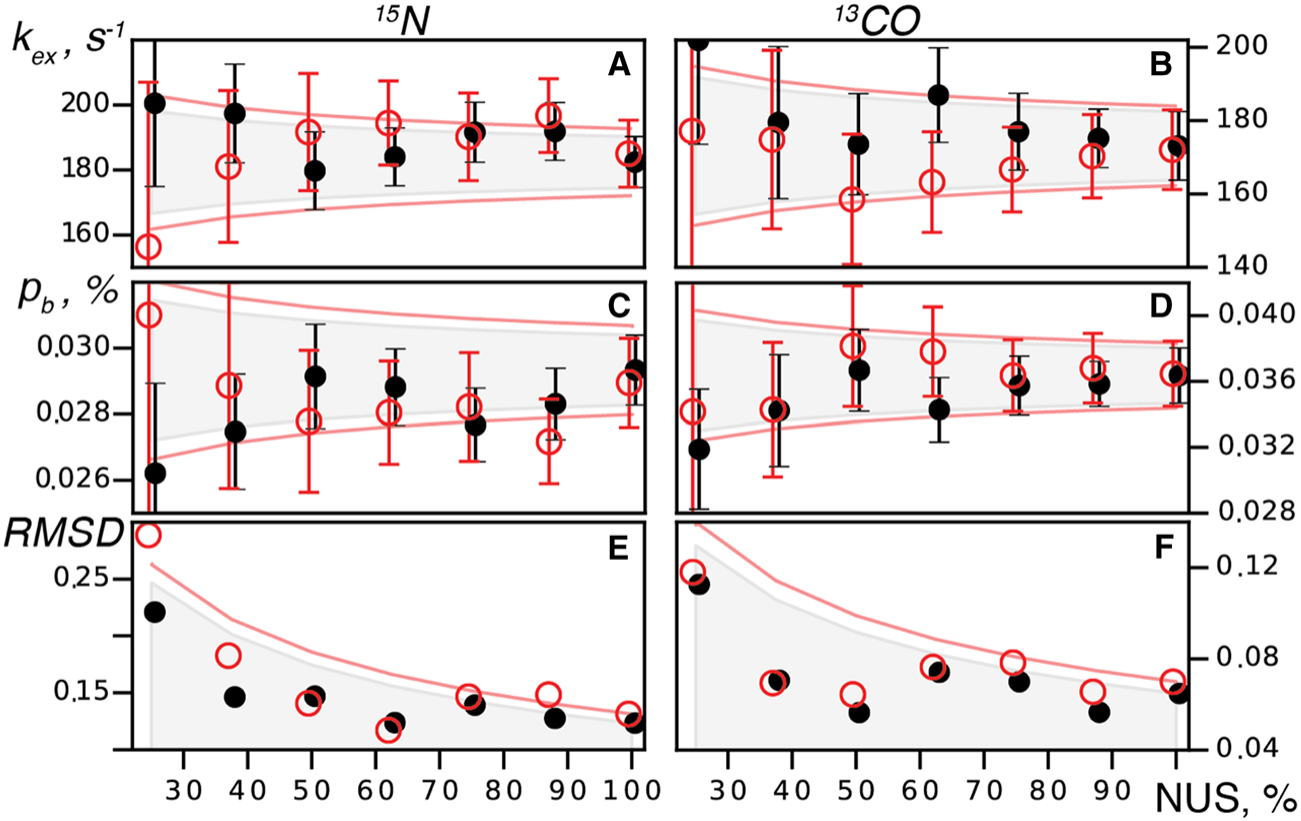
Analysis of the 2D ^15^N and ^13^CO RD experiments on Abp1p SH3 domain partially bound to the Ark1p peptide. Global parameters k_ex_ (**a**,** b**) and p_B_ (**c**,** d**) obtained from the RD as well as RMSD (**e**, **f**) between the Δϖ measured directly and derived from RD are shown versus the spectrum sparse level. Shown is a typical result obtained for a NUS scheme (random seed, flat random distribution) using different estimates of errors for the *R*_*2*_ values: (*black*) from the duplicate measurement and (*red*) from 20 jackknife resampling trials, respectively. *Circles* and *error bars* give fitted values and uncertainties of k_ex_ and p_B_ of the parameters. The areas indicated by *gray color* and restricted by the *red lines* show an anticipated error obtained as an extrapolation of the uncertainty in the reference spectrum to shorter measurement times as ~1⁄√t

First we validated our quantitative NUS spectra reconstruction approach for the traditional 2D versions of the RD experiments obtained for SH3 domain from the yeast protein Abp1p, partially bound to a peptide from the protein Ark1p. Binding of a ligand with *K*_*d*_ = 4.4 μM (Haynes et al. 2007) and *k*_*ex*_ = *k*_*on*_[L] + *k*_*off*_ manifests as CPMG dispersions for various nuclei for a subset of protein residues (Lundstrom et al. 2008, 2009a, b; Hansen et al. 2008a). Furthermore, the difference in chemical shifts between the free and bound states can be measured directly from peak positions in spectra of free and saturated SH3 domain. For a partially bound sample, this allows to not only compare determined values for *k*_*ex*_ and *p*_*B*_ for different pulse sequences but also how accurately chemical shifts of the excited state are determined. Figure 3 and Table 3 demonstrate comparison of the dynamic parameters *p*_*B*_, *k*_*ex*_ and Δϖ obtained from two-dimensional ^13^CO and ^15^N RD experiments recorded in full and with NUS. The NUS spectra were obtained by randomly sub-sampling the fully sampled reference spectra at different sparse levels. Figure 3 shows that in our two-dimensional RD experiments, reliable parameters of the millisecond dynamics can be obtained using down to 25% sparse sampling. This result is in line with recent applications of co-processing to 2D relaxation data (Linnet and Teilum 2016). The observed increase in the error of the dynamic parameters as NUS gets sparser is within the limits expected for the square-root dependence of the spectral signal-to-noise ratio on the measurement time experiments. Thus, the use of NUS and co-MDD processing does not introduce noticeable bias or additional noise into the analysis.

**Table 3:** Summary of relaxation dispersion experiments performed for Abp1p SH3 domain partially bound to Ark1p peptide

Parameter	^15^N CPMG			^13^CO CPMG		
	2D	NUS 2D	NUS 3D	2D	NUS 2D	NUS 3D
Sparseness (%)	100	50	8.3	100	50	5
Acquisition time (h)	18	9	43	16	8	43
Number of residues with significant chemical exchange	14			17		
Exchange rate (s^−1^)	182 ± 8	200 ± 15	211 ± 30	173 ± 9	171 ± 12	170 ± 23
Population of the excited state (%)	2.9 ± 0.1	2.7 ± 0.2	2.8 ± 0.3	3.6 ± 0.2	3.6 ± 0.2	3.5 ± 0.4
RMSD between |Δ*ϖ*_CPMG_| and |Δ*ϖ*_*direct*_| (ppm)	0.12	0.15	0.22	0.07	0.07	0.06

For 50% NUS 2D reported values correspond to mean values/errors over 20 resampling trials. For NUS 2D and 3D datasets errors were estimated via jackknife resampling while for the reference, fully sampled 2D experiments errors were estimated traditionally as a global error from duplicate measurements. NUS 2D spectra were processed with co-RMDD, 3D spectra were processed with co-MDD

### Accurate relaxation parameters from 3D NUS RD experiments

In order to validate the new 3D NUS RD experiments, they were tested for two different proteins and the derived dynamic parameters were compared with the results from the standard 2D experiments. For the disordered cytosolic domain of CD79A chain from the B-Cell receptor, the RD profiles in 2D and 3D experiments were flat. When comparing fits of the RD data to the models with and without conformational exchange, we did not find millisecond dynamics at a significance level of p < 0.01 for any individual amino acid residues, and hence, proceeded with comparing the pairwise root-mean-square-deviation (RMSD) between the experimental data and the best fit to a constant function for the 3D NUS and the standard 2D experiments. The average over all NH group RMSD values for the three- and two-dimensional ^15^N RD experiments were 0.35 ± 0.19 s^−1^, and 0.19 ± 0.09 s^−1^, respectively. Figure S1 shows ^15^N relaxation dispersions for the residues with the smallest, the median and the largest RMSD for the 3D NUS ^15^N RD experiment and the same residues in the standard 2D experiment. Even the highest value of 1.1 s^−1^ for residue A15 is tolerable and the conclusion is that NUS in the three-dimensional pulse sequences does not introduce artefacts into CPMG RD profiles. Clearly, the new experiments can provide just as good precision as the well-established 2D experiments while greatly improving the peak resolution.

**Fig. 4:**
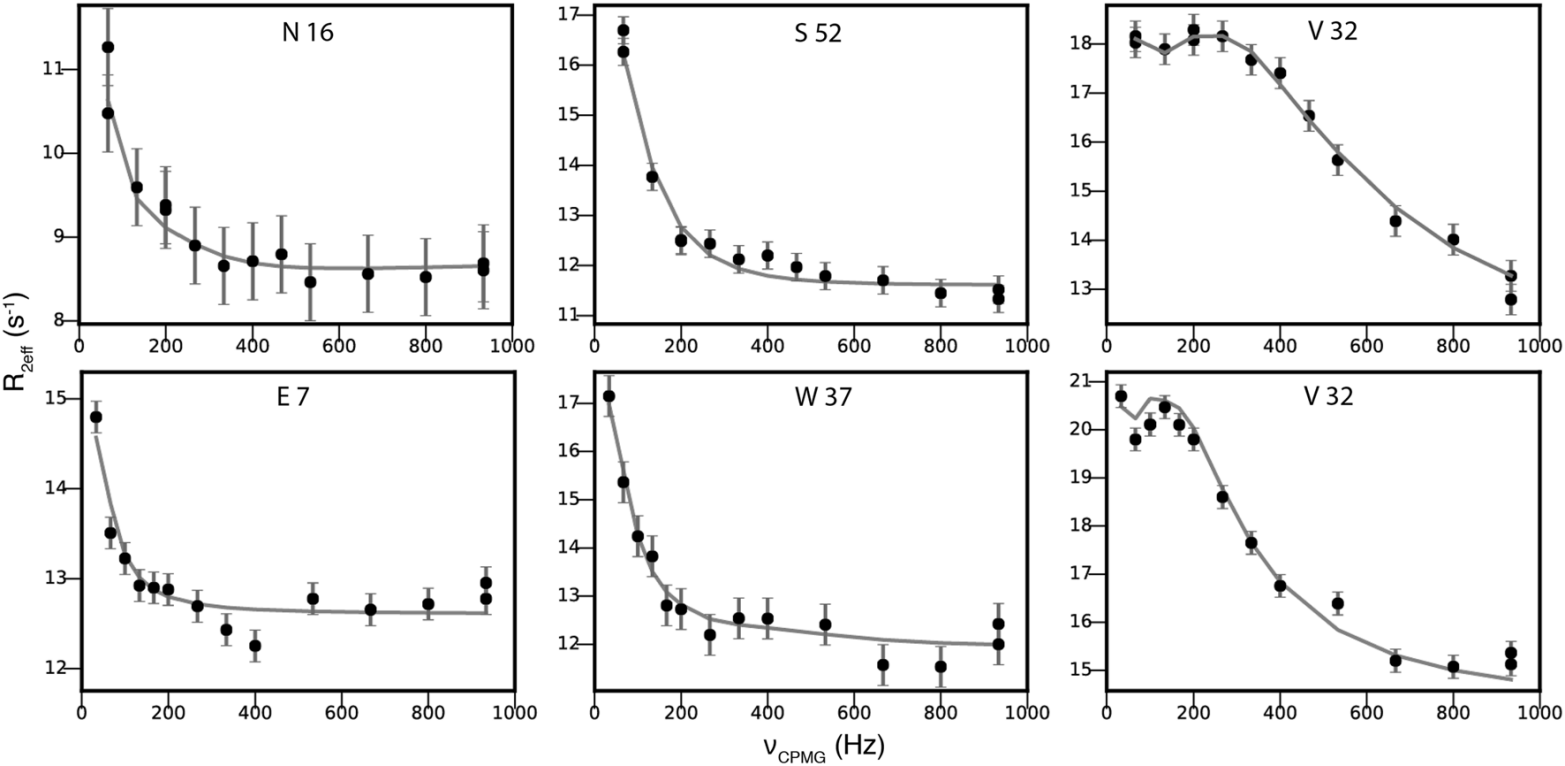
^15^N (*top row*) and ^13^CO (*bottom row*) relaxation dispersion profiles from the 3D NUS experiments for serval residues of Abp1p SH3 domain partially bound to a peptide from Ark1p. The residues with the smallest (N16/E7), median (S52/W37) and largest (V32) |Δϖ| are shown. *Filled circles* represent experimental data collected using the three-dimensional pulse sequence with sparse sampling at 18.8 T. The *line* represents the best fit to a global two-state model

The analysis of the relaxation dispersions of the Abp1p SH3 domain with partly bound Ark1p peptide demonstrated that the new 3D NUS RD experiments are well suited for studies of millisecond dynamics. Table 4 summarizes and compares the results of all experiments when fitted to a global two-state model and Fig. 4 shows that ^15^N as well as ^13^CO experimental data are described well by this model. The global parameters, *p*_*B*_, and *k*_*ex*_, are identical within error regardless of either 2D or 3D experiment was used to probe the dynamics. Small difference between the results obtained from ^15^N and ^13^CO may be explained by apparent coupling between *p*_*B*_, and *k*_*ex*_, parameters.

**Table 4:** Comparison of relaxation dispersion parameters derived for Abp1p SH3 domain partially bound to Ark1p peptide using co-processing with MDD, IRLS-VE methods of NUS spectra reconstruction

Parameter	^15^N CPMG				
	2D^a^	3D co-MDD		3D IRLS-VE	
		Dup^a^	JK^b^	Dup	JK
Exchange rate (s^−1^)	182 ± 8	206 ± 20	211 ± 30	238 ± 28	246 ± 27
Population of the excited state (%)	2.9 ± 0.1	2.9 ± 0.2	2.8 ± 0.3	2.6 ± 0.2	2.6 ± 0.2
RMSD between |Δϖ_cpmg_| and |Δϖ_direct_| (ppm)	0.12	0.19	0.22	0.24	0.26

Parameter	^13^CO CPMG				
	2D^a^	3D co-MDD		3D IRLS-VE	
		Dup^a^	JK^b^	Dup	JK
Exchange rate (s^−1^)	173 ± 9	154 ± 11	172 ± 23	190 ± 12	162 ± 20
Population of the excited state (%)	3.6 ± 0.2	3.7 ± 0.2	3.4 ± 0.4	3.2 ± 0.2	3.7 ± 0.4
RMSD between |Δϖ_cpmg_| and |Δϖ_direct_| (ppm)	0.06	0.06	0.05	0.05	0.05

^a^ *R*_2,*eff*_ errors are estimated as per residue errors derived from duplicate measurements

^b^ *R*_2,*eff*_ errors are estimated via jackknife resampling

We have previously noted that the ^13^CO dispersion profiles for Asp/Asn residues may deviate from the expected appearance and shown that this is due to an unrefocused coupling with the side-chain ^13^CO during the relaxation delay (Lundstrom et al. 2008). When an increasing number of refocusing pulses is applied, the coupling regime changes from weak towards strong, implying that *R*_*2,eff*_ is modulated by ν_CPMG_ even in the absence of chemical exchange. Since the coupling constant is dependent on the χ^1^ dihedral angle, the effect is not equally serious for all residues of these types. We have included an option to refocus the coupling at the expense of slightly lowered sensitivity (Lundstrom et al. 2008) but chose to not use this refocusing element here. Plots for all residues showing the relaxation dispersions are found in Supplementary Figure S2.

**Fig. 5:**
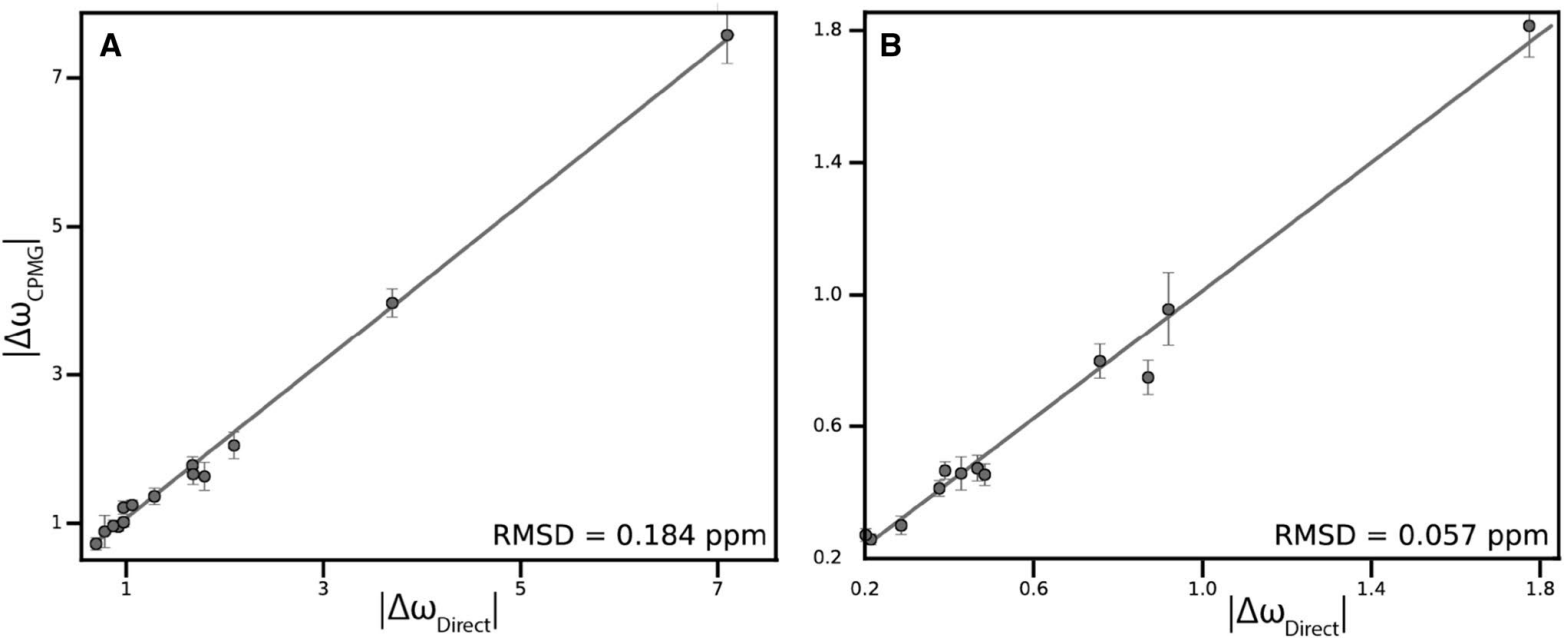
Correlation between the magnitude of difference in chemical shift obtained from the 3D RD experiments (**a** 3D ^15^N-NUS-CPMG and **b** ^13^CO-NUS-CPMG) and calculated from free Abp1p SH3 domain and SH3 domain saturated with Ark1p peptide. All residues with significant chemical exchange are shown. Pairwise RMSD between |Δω_CPMG_| and |Δω_Direct_| are shown in the *bottom-right corners* of the panels. The *straight line* represents |Δω_CPMG_|=|Δω_Direct_| to guide the eye

Lastly, we compared |Δϖ| extracted from the fits of 3D RDs and those measured from the difference in the peak positions in the spectra of free SH3 domain and SH3 domain saturated with Ark1p peptide. Figure 5, demonstrates excellent correlations for both ^15^N and ^13^CO |Δϖ|. For ^13^CO, the pairwise RMSD between the values are equally good for the three-dimensional and two-dimensional experiments. For ^15^N, the values determined from the 2D experiment agree somewhat better with the RMSDs of 0.12 and 0.19 ppm, respectively.

### Comparison of RD’s obtained with co-MDD and IRLS-compressed sensing algorithms

For the processing of sparsely sampled three-dimensional RD experiments we compared Multi-Dimensional Decomposition co-processing (co-MDD) using Eq. 1, and a representative compressed sensing algorithm—Iteratively Reweighted Least Squares with Virtual-Echo enhancement (IRLS-VE) (Mayzel et al. 2014; Kazimierczuk and Orekhov 2011). Results are summarized in Table 4. For the ^13^CO relaxation dispersion experiment both co-MDD and IRLS-VE showed comparable and correct within experimental error results, although the exchange rate error and |Δ*ϖ*| correlations for the IRLS reconstruction were notably higher. For the ^15^N relaxation dispersion experiment, IRLS-VE and co-MDD correspond to each other, although IRLS-VE again gives slightly elevated errors compare to co-MDD processing. Furthermore, comparison with the reference relaxation parameters, derived from fully sampled two-dimensional pulse sequences, shows that IRLS-VE leads to a slightly augmented value of the exchange rate and understated value of the excited state population.

It was important to check how robust the 3D NUS experiments are in respect to the amount of NUS points and if it was possible to further reduce the measurement time. Table 5 depicts the results of the ^15^N RD analysis obtained using co-MDD and IRLS-VE at different NUS levels. Co-MDD produces correct results down to 4.1% NUS level with only a small increase of the errors. IRLS-VE also works although the errors are notably higher and rapidly increase as the NUS level decreases.

**Table 5:** Comparison of relaxation dispersion parameters derived from 3D ^15^N relaxation dispersion experiment on Abp1p SH3 domain partially bound to Ark1p peptide using co-MDD and CS-VE methods at 8.3, 7.0, 5.8 and 4.2% NUS levels

Parameter	^15^N CPMG							
	co-MDD				IRLS-VE			
	8.3%	7.0%	5.8%	4.1%	8.3%	7.0%	5.8%	4.1%
Exchange rate (s^−1^)	211 ± 30	206 ± 20	206 ± 23	209 ± 28	246 ± 27	235 ± 30	243 ± 35	274 ± 54
Population of the excited state (%)	2.8 ± 0.3	2.9 ± 0.2	2.9 ± 0.3	2.8 ± 0.3	2.5 ± 0.2	2.6 ± 0.3	2.6 ± 0.3	2.5 ± 0.4
RMSD between |Δϖ_cpmg_| and |Δϖ_direct_|(ppm)	0.22	0.22	0.24	0.24	0.26	0.25	0.26	0.3

Except for 8.3% NUS, reported values and errors correspond to mean and standard deviation of the related parameters respectively over 20 resampling trials. R_2_ errors at all NUS levels were estimated via jackknife resampling

From this study and from reports of other groups (Long et al. 2015; Linnet and Teilum 2016), we conclude that co-MDD and related methods that simultaneously process spectra corresponding to all CPMG frequencies perform better than the compressed sensing algorithms, which are the most successful when processing single spectra.

### Estimation of R_2_ errors with jackknife approach

Correct estimation of the precision of the relaxation rates in the RD experiments is crucial for accurate calculation of dynamic parameters and their uncertainties. The commonly used approach is to perform duplicate measurements of the relaxation rates for several CPMG frequencies and to derive the error estimates from the variance of the obtained R_2_ values. In this work, we present an alternative approach based on the jackknife resampling. By randomly omitting a fraction (10–20%) of the NUS data, we produce multiple sufficiently independent spectra realizations, from which intensity errors can be obtained for each peak. Figure 3 and Table 4, show that accuracy and precision of the fitted dynamic parameters *k*_*ex*_, *p*_*B*_, and Δϖ obtained from the traditional duplicate measurements and by the jackknife procedure are very similar. This validates the jackknife approach and renders the repeated measurements in NUS spectra unnecessary. Omitting the repeated measurements allows to further reduce time of the RD experiment or to sample of more CPMG frequencies for improving reliability of the analysis.

### Targeted acquisition approach to real-time R_2_ error evaluation

**Fig. 6:**
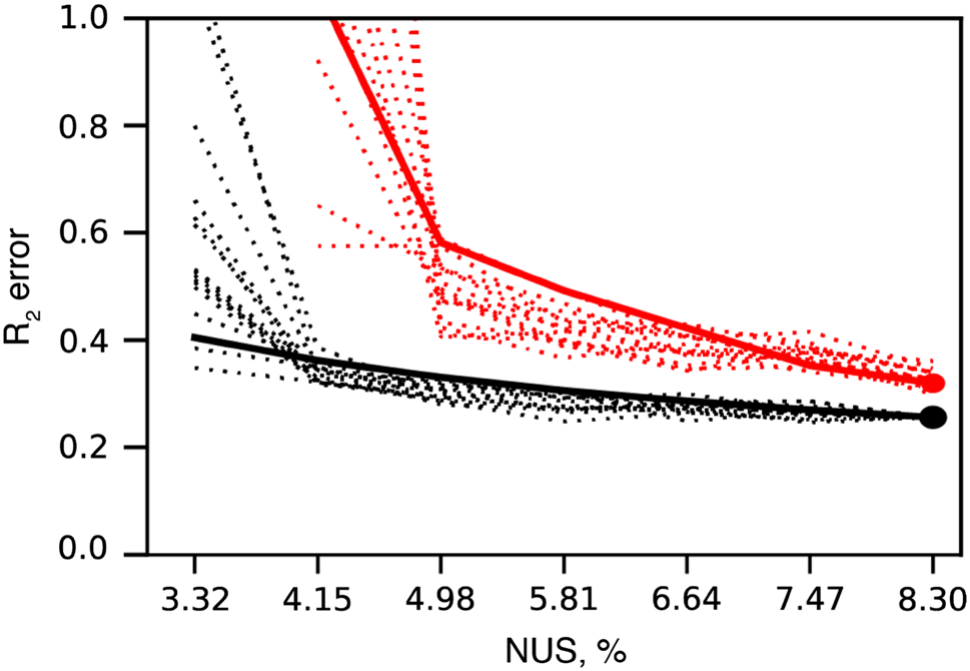
R_2_ error estimation with Targeted Acquisition. TA calculations were performed post hoc by subsampling the 8.3% NUS three-dimensional ^15^N relaxation dispersion experiment on Abp1p SH3 domain partially bound to a peptide from Ark1p. Spectra were processed and analyzed in steps starting from 3.3% NUS, at each step 1% NUS points was added to the final 8.3% NUS. 15 random TA realizations were made to average the effect of various random seeds. *Black dotted lines* and *black dot* at 8.3% NUS correspond to R_2_ error estimated as a global error from duplicate measurements, *black dashed line* corresponds to global error from duplicate measurements at 8.3% NUS scaled according to the measurement time. The *red dot* at 8.3% NUS correspond to R_2_ error estimated from jackknife resampling. *Red dotted lines* correspond to R_2_ error calculated as a variation of R_2_ values on consecutive TA steps. Red solid line corresponds to R_2_ error calculated via jackknife resampling on every TA step

One of the advantages of sparse data acquisition is that the spectra can be processed at any time during acquisition. As a consequence, it is possible to estimate spectrum quality in real-time during the experiment. Depending on the task, various parameters like desired number of peaks, peak intensity or R_2_ error, as in the current study, can be set as an experiment ‘target’ in the procedure that we call Targeted Acquisition (TA) (Isaksson et al. 2013; Jaravine and Orekhov 2006; Jaravine et al. 2008). Errors in intensity and R_2_ for a peak can be estimated as the variation between the values at consecutive moments of data collection, e.g. between 4 and 5% NUS. The TA approach can be thought as a proxy of the resampling method with only a single resampling event. In order to improve the statistics, we calculate an average intensity error over multiple spectral peaks. This should be distinguished from the true jackknife resampling, where intensity errors of individual peaks are obtained from the statistical analysis over multiple resampling trials. Figure 6 demonstrates and compares various approaches for TA error estimation, where black lines correspond to the traditional error estimation from duplicate measurements, red dashed lines correspond to variation of R_2_ values between consecutive TA steps, and red solid line corresponds to the jackknife approach for R_2_ error estimation. As can be seen from the black curves the R_2_ error shoots up at 4.15% NUS. This is the NUS level, where there is simply not enough data for good spectra reconstruction by co-MDD. As both TA and jackknife approaches relies on subsampling, their R_2_ errors estimates depend on the spectrum quality at 10–20% lower NUS levels. This explains why the R_2_ errors obtained from TA and jackknifes shoot up at 5% NUS and have somewhat higher, i.e. by less than 30%, values relative to the errors obtained from the duplicate measurements. R_2_ errors obtained by all three methods are comparable, which allows use of the more practically convenient jackknife as well as validates the TA approach for quantitative monitoring of the spectrum quality improvement in real time during the experiment.

## Conclusions

In this work, we introduce a new approach for acquisition and processing of the relaxation dispersion experiments for the protein backbone ^15^N and ^13^CO atoms. The main advantage of the new method is the much-improved spectral resolution, which allows characterization of protein dynamics of those peaks, that overlap in the traditional spectra. We present two new 3D pulse sequences for ^15^N and ^13^CO RD experiments. In order to keep the measurement time of the high resolution 2D and 3D experiments short and comparable to the duration of the traditional 2D experiments, we use NUS. We show that the best accuracy and precision of the derived parameters of the conformational exchange are obtained when the NUS spectra corresponding to the individual CPMG frequencies are co-processed using multi-dimensional decomposition. Quantitative analysis of the spectra processed individually with the compressed sensing is also possible, although the results are noticeably worse. In order to further reduce the measurement time, we introduce a new method for estimation of errors in the relaxation rates. Namely, we suggest to replace the time consuming repeated measurements with the jackknife resampling of the NUS data. In practice, it may be difficult to predict required experimental time and NUS level needed to achieve acceptable precision of the relaxation rates for a signal of interest. We show that estimates of the precision may be obtained during the experiment in real time, thus allowing to “target” the RD experiment for a predefined precision. The error estimates obtained from the jackknife resampling and targeted procedure are similar to the errors derived from the traditional approach with the duplicate measurements.

## Electronic supplementary material

Below is the link to the electronic supplementary material.

Supplementary material 1 (DOCX 1068 KB)
