# Supplementary material for: Measurement of protein backbone 13CO and 15N relaxation dispersion at high resolution
Source: J Biomol NMR. 2017 Sep 1;69(1):1–12. doi: 10.1007/s10858-017-0127-4 (PMC5626786; doi:10.1007/s10858-017-0127-4)
Supplement: Supplementary file 1 — Supplementary material 1 (DOCX 1068 KB) [file 10858_2017_127_MOESM1_ESM.docx]

^
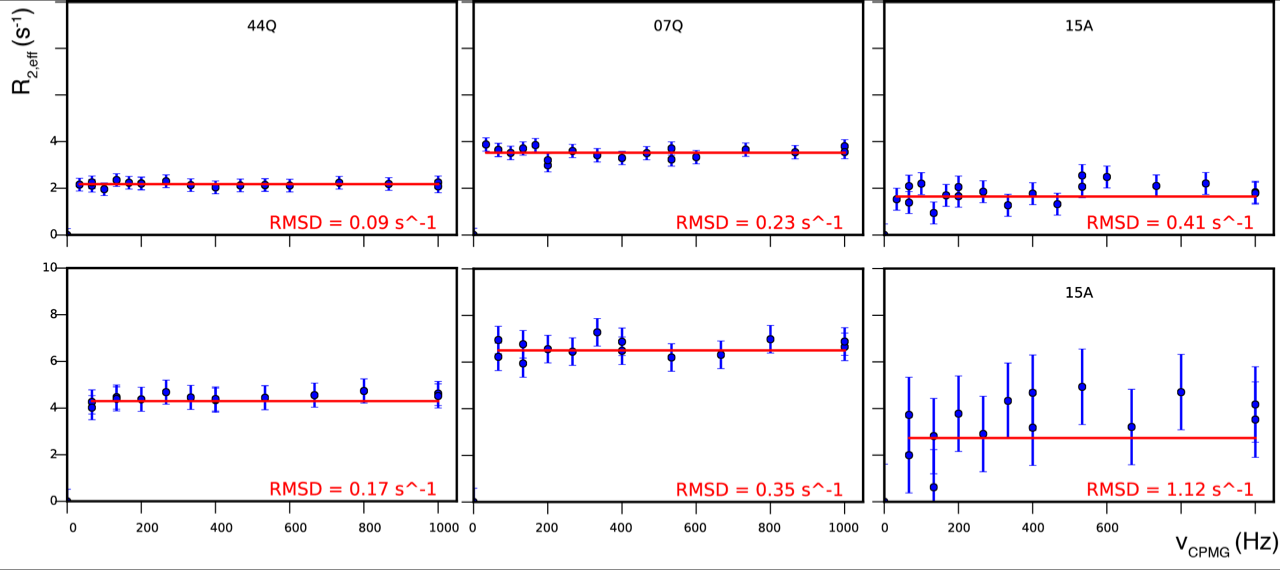
^

**Figure S1.** ^15^N effective transverse relaxation rates from the 3D NUS experiments for CD79a as a function of the effective field. Results for: **Top row)** standard two-dimensional experiment and **Bottom row)** NUS three-dimensional experiment. The filled circles represent experimental data and the line represents the best fit to a constant function. The RMSD between experimental data and the fitted function is indicated in the figure. Data for the residues with the lowest (Q44), median (Q7) and highest (A15) RMSD in the three-dimensional experiment is shown. The reason for the different of R_2,eff_ values in the two classes of experiments is that the magnetization during the relaxation delay was TROSY in the standard two-dimensional experiment but not in the NUS three-dimensional experiment.

**Figure S2.** ^13^CO relaxation dispersion profiles from the 3D NUS experiments for the residues of Abp1p SH3 domain partially bound to a peptide from Ark1p. Filled circles represent experimental data collected using the three-dimensional pulse sequence with sparse sampling at 18.8 T. The residue numbers refer to the CO groups where the relaxation dispersions were measured.

**Figure S2** (continued)

**Figure S2** (continued)
